# Items

**Published:** 1909-01

**Authors:** 


### ITEMS.

M. J. Breitenbach Company, 53 Warren Street, New York, importers and distributors of Pepto-Mangan (Gude), have sent out their Physician's Daily Memorandum for 1909. This great house has distributed these books to the profession for several years past, and they have proven one of the most useful articles of the kind for the physician's desk ever gratuitously distributed.

---

The Antikamnia Calendar for 1909 presents on the obverse a portrait in colors, of a young girl holding a flower, and is entitled "Purity." On the reverse is the calendar proper, and memoranda relating to antikamnia. It is an exquisite blending of art and business, and will be sent to physicians.

